# Prevalence and Risk Factors for Keratoconus in Young Adults Assessed with Tomography and Corneal Biomechanics: A Prospective Cross-Sectional Study

**DOI:** 10.1016/j.xops.2026.101109

**Published:** 2026-02-06

**Authors:** Lorena Santos Barros, Alexandre Batista da Costa Neto, Louise Pellegrino G. Esporcatte, Marcella Quaresma Salomão, Bruno Frujuelli de Melo, Dillan Cunha Amaral, Renata A. Rezende, Mariana Lopes Souza, Érica Rossi Garcia, Luca Gualdi, Stephen D. Klyce, Aydano Machado, Renato Ambrósio

**Affiliations:** 1 Department of Ophthalmology, Federal University of the State of Rio de Janeiro, Rio de Janeiro, Rio de Janeiro, Brazil; 2 Department of Ophthalmology, Federal University of São Paulo, São Paulo, Brazil; 3 School of Medicine, Federal University of Rio de Janeiro, Rio de Janeiro, Rio de Janeiro, Brazil; 4 Post-Graduate Program in Ophthalmology, Pontifical Catholic University, Rio de Janeiro, Rio de Janeiro, Brazil; 5 Diagnostica Oftalmologica e Microchirurgia Oculare, Rome, Italy; 6 Department of Ophthalmology, Icahn School of Medicine at Mount Sinai, New York, New York; 7 Department of Computer Science, Federal University of Alagoas, Maceio, Alagoas, Brazil

**Keywords:** Keratoconus, Prevalence, Subclinical ectasia, Eye rubbing, Brazil

## Abstract

**Purpose:**

To evaluate the prevalence of keratoconus (KC) and associated risk factors in a previously undiagnosed population at a Brazilian university hospital, using advanced tomographic and biomechanical tools.

**Design:**

A prospective, cross-sectional observational study.

**Methods:**

A total of 521 participants (1041 eyes) from the staff and students of Gaffrée and Guinle University Hospital, Federal University of the State of Rio de Janeiro, were examined using corneal tomography (Pentacam AXL; Oculus Optikgeräte GmbH) and biomechanical analysis (Corvis ST; Oculus Optikgeräte GmbH). Exclusion criteria included self-reported KC, prior corneal surgery, known corneal pathology, and suspected contact lens warpage. A structured questionnaire assessing demographics, eye-rubbing habits, and family history was administered to all participants before diagnostic examinations. A single experienced examiner performed subjective topometric map evaluations. Objective parameters included the Tomographic and Biomechanical Index version 2 (TBIv2), Corneal Biomechanical Index (CBI), Pentacam Random Forest Index, Belin-Ambrósio Deviation Index, version 3 (BAD-Dv3), and topographic KC classification. Statistical analyses included chi-square tests, odds ratios (ORs), and relative risks (RRs). All prevalence rates are reported per eye.

**Results:**

Subjectively, 36.8% of eyes were classified as normal, 31.4% with regular astigmatism, and 5.8% as ectatic or ectasia-suspect (including 4.2% suspected KC, 1.0% KC, and 0.5% pellucid marginal degeneration). Contact lens warpage (15.1%) and other irregular patterns (10.9%) were also noted. Objectively, 5.86% had altered topographic KC classification, 13.16% had pachymetry <500 μm, 20.6% showed Ambrósio relational thickness maximum <385, 10.9% had BAD-Dv3 ≥1.9, 26.9% had CBI ≥0.5, and 32.7% had TBIv2 ≥0.43, with 13.6% ≥0.8. Eye rubbing was significantly associated with KC (OR 20.34; RR 9.79).

**Conclusions:**

This study revealed a notable prevalence of undiagnosed clinical and subclinical KC, with eye rubbing as a strong behavioral risk factor. Multimodal diagnostics allowed more precise characterization of corneal biomechanical susceptibility. The clinical findings support the importance of early screening and behavioral education.

**Financial Disclosure(s):**

Proprietary or commercial disclosure may be found in the Footnotes and Disclosures at the end of this article.

Keratoconus (KC) is a corneal condition characterized by progressive thinning and conical deformation. It can lead to irregular astigmatism, myopia, and impaired visual acuity.^1^^,^^2^ Typically beginning in adolescence or early adulthood, its progression varies and is influenced by a complex interplay of genetic, environmental, and biomechanical factors, including genetic predispositions, hormonal imbalances, eye rubbing, and exposure to ultraviolet light.^3^^,^^4^ Structural changes in KC, including degradation of Bowman layer and stromal collagen due to increased proteolytic activity, compromise corneal integrity and lead to the characteristic conical shape.^4^^,^^5^

Several studies in the medical literature highlight that the prevalence of KC varies significantly across different regions and populations.^6^ Variations in reported KC prevalence may stem from differences in diagnostic tools and age groups. A classic 53-year United States study by Kennedy et al^7^ reported a prevalence of 0.05% (54.5 per 100 000 population), using limited methods such as scissor retinoscopy and irregular keratometry, which only detect advanced cases but remain widely cited. Differences in diagnostic tools, age groups, and demographics likely explain the variation in study results. A Saudi Arabian study found a prevalence of 4.79% among pediatric patients, much higher than in other areas.^8^ Existing research often lacks representation from diverse populations, particularly in areas like South America, and overlooks behavioral and environmental risk factors, such as eye rubbing, in prevalence estimates.^9^^,^^10^

Gaps in KC diagnosis can lead to delayed treatment and worse outcomes.^4^^,^^11^ The aim of this study was to evaluate the prevalence of KC and ectatic susceptibility in a Brazilian university hospital population with no prior diagnosis, using advanced tomographic and biomechanical indices, including research-grade artificial intelligence–derived metrics intended to support, but not replace, clinical judgment, and to assess associated risk factors, particularly family history and eye rubbing. By addressing an understudied population, this research offers valuable insights into the epidemiology of KC within a diverse demographic context. It highlights the need for objective diagnostic indices to reduce diagnostic variability.

## Methods

### Study Design and Ethical Approval

We conducted a prospective, cross-sectional, observational study at the Department of Ophthalmology, Gaffrée and Guinle University Hospital, Federal University of the State of Rio de Janeiro (UNIRIO). The study was approved by the UNIRIO Research Ethics Committee (Certificate of Submission for Ethical Review: 58513822.4.0000.5258), and all participants provided written informed consent. Because only individuals aged ≥20 years were included, parental consent was not required. The study population consisted of approximately 3000 university members.

### Inclusion and Exclusion Criteria

The target population consisted of approximately 3000 university members aged ≥20 years, including students, residents, faculty, and administrative staff. Inclusion criteria required being ≥20 years old, affiliated with UNIRIO, and able to undergo both corneal tomography and biomechanical assessment, as well as to complete the structured ocular health questionnaire. Exclusion criteria comprised a prior diagnosis of KC or pellucid marginal degeneration, corneal ectasia secondary to trauma or infection, and any previous corneal surgical procedures such as refractive surgery, pterygium excision, intrastromal corneal ring implantation, or corneal cross-linking. Eyes with incomplete examinations or imaging labeled as low quality by device quality metrics were also excluded.

### Clinical Examination and Data Collection

**Figure 1:**
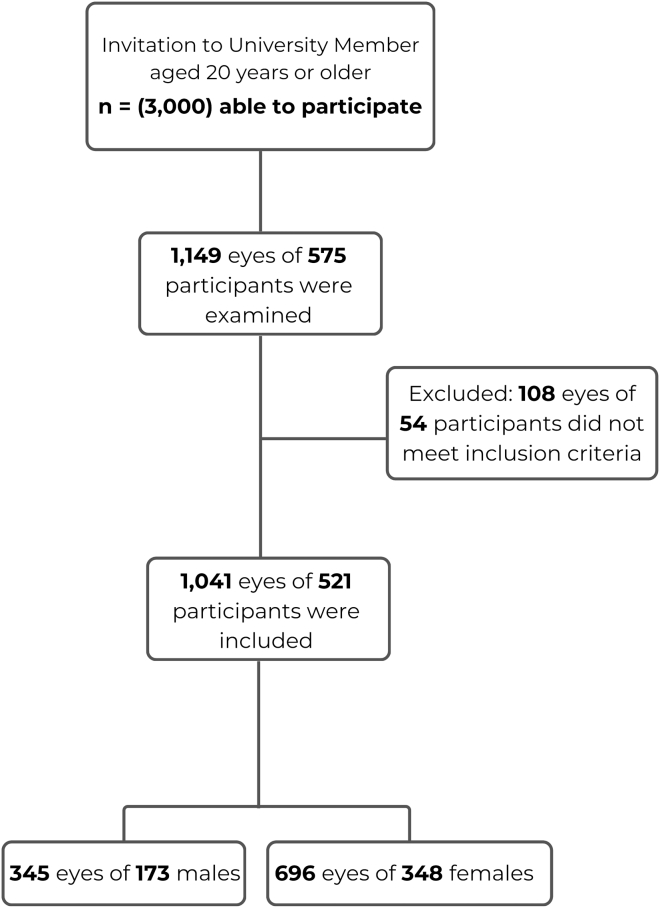
Study design flowchart of included patients.

A total of 1149 eyes from 575 participants were initially examined. After the application of the exclusion criteria, 1041 eyes from 521 participants (520 binocular and 1 monocular) were included in the final analysis (Fig 1). Demographic data (age, sex, family history of KC, and eye rubbing), diagnostic methods (corneal tomography using Pentacam Axial Length (AXL) and biomechanical analysis with Corneal Visualization Scheimpflug Technology (Corvis ST), and participant-reported information through structured questionnaires were collected. The questionnaire assessed ocular health history, including prior diagnoses, surgeries, allergies, and eye-rubbing habits. This questionnaire link may be accessed in the Supplemental File 1 (available at www.ophthalmologyscience.org).

One experienced ophthalmologist (L.B.) also performed all examinations. The Pentacam is based on Scheimpflug imaging, which enables the creation of a 3-dimensional model of the cornea and anterior chamber. On the other hand, the Corvis ST utilizes an air-puff tonometry method combined with high-speed Scheimpflug imaging to assess the biomechanical properties of the cornea.^12^ Only high-quality measurement results with “OK” or acceptable quality scores were used in our analysis.

### Participant Referral and Education

Following testing in line with ethical guidelines, patients with suspicious corneal findings identified by the examiner were offered referral information for cornea specialists at UNIRIO. However, no diagnostic or therapeutic interventions were conducted within the scope of this study. The study provided participants with information about KC as part of the Violet June awareness campaign.^13^ Participants were also educated about KC prevention, with a particular emphasis on the importance of avoiding eye rubbing.

### Subjective Topographic Classification

After the examinations were completed, specific anterior topographic maps from Pentacam were generated and provided to a single experienced examiner (S.D.K.). The examiner assessed the anterior topometric axial (or sagittal) curvature maps for descriptive purposes. We recognize that this approach predates the Global Consensus (2015) recommendations, which have been criticized.^14^^,^^15^ Therefore, these classifications were used solely for morphological comparison and were not considered diagnostic. The examiner conducted a subjective classification of the maps according to the following nomenclature: normal, astigmatism, suspected KC (suspicious patterns that lacked definitive objective confirmation), KC, pellucid marginal degeneration, contact lens warpage (irregular astigmatism requiring exclusion of lens warpage), myopic refractive surgery, hyperopic refractive surgery, and other (other/poor quality, irregularity after cataract surgery, penetrating keratoplasty, or inability to classify).

### Objective Tomographic and Biomechanical Parameters

The prevalence of each classification was calculated based on the number of affected eyes and individuals for the examiner. In addition, an objective analysis of parameters generated by the examinations was performed to calculate prevalence based on the following metrics: mean keratometry (Mean K), defined as the average of flat and steep keratometry readings (K1 + K2/2); the absolute value of the surface variation index over 6 mm (S-Abs 6 mm); the KC percentage index (KISA%); maximum keratometry; topographic KC classification; minimum pachymetry at the thinnest point (Pachy Min); Ambrósio relational thickness maximum value (ARTmax); Belin-Ambrósio enhanced ectasia display version 3 (BAD-Dv3); Pentacam Random Forest Index (PRFI); Corneal Biomechanical Index (CBI); and Tomographic and Biomechanical Index version 2 (TBIv2).

Diagnostic thresholds for the indices applied in this study were prespecified according to the multicenter validation study by Ambrósio et al,^16^ which optimized and externally validated tomographic and biomechanical indices in 3886 eyes from 3412 participants across 25 international centers.

In that analysis, receiver operating characteristic curves were used to maximize the Youden index (sensitivity + specificity – 1), and the area under the curve (AUC) with 95% confidence intervals (CIs) was reported to quantify diagnostic accuracy.

For the Tomographic and Biomechanical Index version 1 (TBIv1), optimal thresholds of 0.50 for clinical ectasia (AUC = 0.986 [95% CI, 0.982–0.990]; sensitivity 98.5%, specificity 98.6%) and 0.29 for very asymmetric ectasia with normal topography (AUC = 0.941 [0.931–0.950]; sensitivity 76.0%, specificity 89.1%) were established. The optimized TBIv2 algorithm, integrating 18 tomographic and biomechanical parameters through random forest modeling, showed superior diagnostic performance with AUC = 0.999 (95% CI, 0.997–1.000) for clinical ectasia (cutoff ≥0.80; sensitivity 98.7%, specificity 99.2%) and AUC = 0.945 (95% CI, 0.935–0.954) for very asymmetric ectasia with normal topography (cutoff ≥0.43; sensitivity 84.4%, specificity 90.1%). Similarly, the BAD-Dv3 thresholds of ≥1.98 for clinical ectasia (AUC = 0.986 [95% CI, 0.982–0.990]; sensitivity 96.8%, specificity 99.3%) and ≥1.27 for very asymmetric ectasia with normal topography (AUC = 0.876 [0.860–0.892]; sensitivity 70.8%, specificity 80.4%) were employed. The PRFI also demonstrated high diagnostic accuracy (AUC = 0.972 [95% CI, 0.967–0.977]) in the same dataset, with cutoffs optimized to maximize the Youden index in distinguishing normal eyes from KC/very asymmetric ectasia cases. The detailed diagnostic accuracy metrics and optimal cutoff values for all indices are summarized in Table S1 (available at www.ophthalmologyscience.org).

Additional indices, including the CBI, ARTmax, inferior–superior value, and maximum keratometry, were applied according to validated reference thresholds balancing sensitivity and specificity (e.g., inferior–superior value ≤1.45 D to exclude significant corneal asymmetry).^16^

### Statistical Analysis

Using a formula for finite populations with a 5% sampling error and a 95% confidence level, the required sample size was calculated to be 342 individuals from the approximately 3000 individuals able to participate in this research. To account for the correlation between the 2 eyes of the same subject, data were analyzed using generalized estimating equations. An exchangeable (compound symmetry) working correlation structure was assumed, considering both eyes equally correlated within each subject. No additional covariates were included in the model. Robust (sandwich) standard errors were applied to obtain consistent parameter estimates and CIs, independent of potential misspecification of the working correlation.

Homogeneity testing, such as the chi-square test, was applied to determine whether statistically significant differences existed in the proportions of patients diagnosed with KC who reported eye-rubbing behavior across the evaluators. Furthermore, odds ratios (ORs) and relative risks (RRs) were calculated to assess the likelihood and risk associated with eye-rubbing behavior among diagnosed individuals.

The overall sample was characterized according to sociodemographic and clinical variables, including gender, age, and key diagnostic indicators of KC. The data were analyzed using absolute and relative frequencies, with emphasis on diagnostic markers such as the CBI, PRFI, TBIv2, and BAD-Dv3. Moreover, associations between participant subgroups were examined, particularly in relation to behavioral factors such as eye rubbing and a family history of KC. Comparative analyses were also conducted between male and female participants to investigate potential gender-related differences in the evaluated parameters.

## Results

Of the 521 participants (1041 eyes), 173 (33.2%) were male and 348 (66.8%) were female. The average age was comparable between genders, with males averaging 38.32 ± 13.97 years and females 38.85 ± 13.18 years. Overall, the mean participant age was 38.83 ± 13.85 years.

Table 1 shows the absolute and relative frequencies of the variables analyzed in the study. These variables were derived from diagnostic tests assessing corneal health, including keratometric indices, pachymetry, and biomechanical measurements. The respective thresholds for these parameters were determined based on previous studies.^16^^,^^17^ Among the findings, altered topographic KC classification was identified in 5.8% of eyes, while 13.1% had minimum pachymetry values of <500 μm. Indicators suggestive of ectatic risk were also notable: 20.6% of eyes had an ARTmax of <385, 10.9% presented with a BAD-Dv3 score of ≥1.9, and 26.9% showed a CBI of ≥0.5. In addition, 13.45% of the sample met the TBIv1 criteria with a score of ≥0.5, and 13.6% had a TBIv2 score of ≥0.8.

**Table 1:** Absolute and Relative Frequencies of Diagnostic Parameters Based on Established Cutoff Values

Variable	Absolute Frequency	Relative Frequency
Sim K Average >47.2∗	11	1.06%
IS Abs 6 mm >1.45	29	2.79%
KISA >60	23	2.21%
Kmax >47.6	29	2.79%
Altered TKC (TKC∗)	61	5.86%
Pachy Min <500 μm	137	13.16%
ARTmax <385	215	20.65%
BAD-Dv3 ≥1.9	114	10.95%
BAD-Dv3 ≥1.6	183	17.58%
PRFI ≥0.38	77	7.40%
PRFI ≥0.15	238	22.86%
CBI ≥0.5	280	26.90%
CBI ≥0.35	442	42.46%
TBIv1 ≥0.5	140	13.45%
TBIv1 ≥0.29	367	35.25%
TBIv2 ≥0.80	142	13.64%
TBIv2 ≥0.43	341	32.76%

ARTmax = Ambrósio relational thickness maximum value; BAD-Dv3 = Belin-Ambrósio enhanced ectasia display version 3; CBI = Corneal Biomechanical Index; KISA% = keratoconus percentage index; Kmax = maximum keratometry; Mean K = mean keratometry (K1 + K2/2); Pachy Min = minimum pachymetry at the thinnest corneal point; PRFI = Pentacam Random Forest Index; S-Abs 6 mm = absolute value of the surface variation index over the central 6 mm; TBIv1 = Tomographic and Biomechanical Index version 1; TBIv2 = Tomographic and Biomechanical Index version 2; TKC∗ = topographic keratoconus classification, categorized as altered (including TKC∗ stages TK1, TK2, TK3, or TK4) or nonaltered (–).

Table 2 presents the absolute and relative frequencies of subjective classifications made by the evaluator based on anterior topometric maps, encompassing a total of 1041 eyes analyzed. Most eyes were classified as normal (36.8%) or as having astigmatism (31.4%). Cases of suspected KC eyes accounted for 4.2%, and confirmed KC represented 1.1% of the total. This pattern recognition was based on curvature irregularity, and so suspected KC represented eyes with suspicious patterns that lacked definitive objective confirmation.^18^ Additionally, 15.1% of eyes were classified as having contact lens warpage, while other classifications, such as pellucid marginal degeneration (0.5%), hyperopic refractive surgery (0.1%), and other (10.9%), were less frequent. Among those diagnosed subjectively with KC, 54.5% of participants reported habitual eye rubbing. In this group, the evaluator calculated an OR of 20.34 and an RR of 9.79, indicating a strong association between eye rubbing and the presence of KC, based on the subjective topometric classification.

**Table 2:** Subjective Classification of Corneal Maps: Absolute and Relative Frequencies of Diagnostic Categories (Expressed in the Number of Eyes)

Classification	Evaluator 1
NRM	383 (36.8%)
KC	11 (1.0%)
KCS	44 (4.2%)
AST	327 (31.4%)
OTH	113 (10.9%)
CLW	157 (15.1%)
PMD	5 (0.5%)
MRS	0 (0.0%)
HRS	1 (0.1%)
Total	1041 (100%)

AST = astigmatism; CLW = contact lens warpage (asymmetric inclination); HRS = hyperopic refractive surgery; KC = keratoconus; KCS = suspected keratoconus; missing: absent data; MRS = myopic refractive surgery; NRM = normal; OTH = other (includes poor image quality, irregularities after cataract surgery, penetrating keratoplasty, or cases unable to be classified); PMD = pellucid marginal degeneration.

Table 3 summarizes the main quantitative parameters obtained from corneal tomography and biomechanical assessments. The mean keratometry was 43.38 ± 1.48 D, and the mean maximum anterior keratometry was 44.45 ± 1.75 D. Corneal astigmatism averaged 1.02 ± 0.84 D. The mean pachymetry at the apex was 542.45 ± 32.71 μm. Regarding ectasia-related indices, the BAD-Dv3 had a mean of 1.03 ± 0.99, and ARTmax was 456.01 ± 90.32. The biomechanical and integrative indices showed mean values of 0.12 for PRFI, 0.23 for TBIv1, 0.34 for TBIv2, and 0.34 for CBI. A significant negative correlation was found between pachymetry at the pupil and CBI values (Spearman rho = –0.74, *P* < 0.001), indicating that higher CBI values were associated with thinner corneas. Mean intraocular pressure was 15.26 ± 2.44 mmHg, and the biomechanically corrected intraocular pressure was 14.81 ± 2.06 mmHg.

**Table 3:** Descriptive Statistics of Tomographic and Biomechanical Parameters

Variable	Mean	Standard Deviation	Minimum	Maximum
Mean K	43.38	1.476	38.600	48.25
Astig	1.02	0.838	0.000	11.60
Axis (flat)	88.76	73.662	0.000	180.00
IS Value absolute	0.53	0.664	0.000	13.05
KISA	14.35	49.662	0.333	1413.52
Kmax (Front)	44.45	1.746	39.140	55.72
Pachy Apex	542.44	32.705	470	645
PachyMinX	0.00261	0.709	–1.280	1.92
ARTmax	456.01	90.316	50	823
BAD-Dv3	1.03	0.991	–1.110	18.55
PRFI	0.12	0.164	0.000	1.00
SPa1	103.76	18.264	62.675	198.95
CBI	0.34	0.25	0.001	1.0
TBIv1	0.23	0.257	0.000	1.00
TBIv2	0.34	0.306	0.000	1.00
IOP [mmHg]	15.26	2.435	7.500	32.00
bIOP	14.81	2.055	7.700	27.00

ARTmax = Ambrósio relational thickness maximum value; Astig = corneal astigmatism magnitude; Axis (flat) = axis of the flat keratometry meridian (in degrees); BAD-Dv3 = Belin-Ambrósio enhanced ectasia display; bIOP = biomechanically corrected intraocular pressure (mmHg); CBI = Corneal Biomechanical Index; IOP = intraocular pressure measured by Corvis ST (mmHg); IS Value absolute = absolute value of the surface asymmetry index; KISA% = keratoconus percentage index; Kmax (Front) = maximum anterior keratometry; Mean K = mean keratometry (K1 + K2/2); Pachy Apex = pachymetry at the corneal apex; PachyMinX = horizontal position (X-axis) of the thinnest corneal point; PRFI (cutoff ≥0.15) = Pentacam Random Forest Index; SPa1 = stiffness parameter at first applanation; TBIv1 = Tomographic and Biomechanical Index version 1; TBIv2 (cutoff ≥0.29) = Tomographic and Biomechanical Index version 2.

Axis (flat) – 0° and 180° are the same axis.

Table 4 presents the distribution of qualitative variables among participants who met the diagnostic cutoff values for the most important tomographic and biomechanical indices related to KC. The data are shown both by total number of eyes and individuals, with further stratification by sex, presence of a family history of KC, self-reported eye-rubbing behavior, and use of eye drops. Among individuals with a CBI score of ≥0.5 (n = 187), 63.1% were women, and more than half reported experiencing eye rubbing (54.5%). A positive family history of KC was present in 31.0%, and 19.2% reported using eye drops. For the PRFI ≥0.38 group (n = 52), 71.1% were women, with eye rubbing reported by 63.4% and a family history present in 36.5%. Among those with TBIv1 ≥0.5 (n = 99), 64.6% were women, and the prevalence of eye rubbing and family history was 59.6% and 35.3%, respectively. Similarly, individuals with TBIv2 ≥0.8 (n = 95) showed a predominance of women (67.4%), as well as high rates of eye rubbing (60.0%) and a family history (33.6%). Finally, for the group with BAD-Dv3 ≥1.9 (n = 72), 66.6% were women, and 62.5% reported eye rubbing, with a family history present in 37.5%.

**Table 4:** Distribution of Participants by Diagnostic Cutoffs and Associated Clinical and Behavioral Factors

Variable	Total Eyes	Total Individuals	Men	Women	Family History of Keratoconus	Eye Rubbing	Use of Eye Drops
CBI ≥0.5	280	187 (35.8%)	69 (36.9%)	118 (63.1%)	58 (31.0%)	102 (54.5%)	36 (19.2%)
PRFI ≥0.38	77	52 (9.9%)	15 (28.8%)	37 (71.1%)	19 (36.5%)	33 (63.4%)	13 (25%)
TBIv1 ≥0.5	140	99 (19.0%)	35 (35.3%)	64 (64.6%)	35 (35.3%)	59 (59.6%)	22 (22.2%)
TBIv2 ≥0.8	142	95 (18.2%)	31 (32.6%)	64 (67.4%)	32 (33.6%)	57 (60.0%)	22 (23.1%)
BAD- Dv3 ≥1.9	114	72 (13.8%)	24 (33.3%)	48 (66.7%)	27 (37.5%)	45 (62.5%)	16 (22.2%)

BAD-Dv3≥1.9 = Belin-Ambrósio enhanced ectasia display ≥1.9; CBI ≥0.5 = Corneal Biomechanical Index ≥0.5; PRFI ≥0.38 = Pentacam Random Forest Index ≥0.38; TBIv1 ≥0.5 = Tomographic and Biomechanical Index version 1 ≥0.5; TBIv2 ≥0.8 = Tomographic and Biomechanical Index version 2 ≥0.8.

The table presents the distribution of individuals and eyes meeting each diagnostic cutoff, segmented by sex, presence of family history of keratoconus, reported eye-rubbing behavior, and use of eye drops.

## Discussion

Keratoconus diagnosis relies on clinical examination and advanced imaging.^19^ Early diagnosis is essential to prevent progression and preserve visual function, as untreated KC can lead to irregular astigmatism, scarring, or even corneal perforation. In our study, the prevalence of KC varied depending on the diagnostic index applied: 13.6% of eyes (18.2% of individuals) using the TBIv2 ≥0.8 and 10.9% of eyes (13.8% of individuals) using the BAD-Dv3 ≥1.9. These findings underscore the impact of diagnostic criteria on prevalence estimates and highlight the need for standardized multimodal screening. Advanced imaging techniques enhance early detection, enabling personalized management to optimize outcomes and minimize the need for invasive interventions.^20^

The primary diagnostic tools for detecting KC include corneal topography,^11^^,^^21^^,^^22^ tomography,^22^, ^23^, ^24^ and biomechanical assessments.^23^^,^^25^^,^^26^ Each provides unique insights into the structural and functional characteristics of the cornea, which are essential for accurate diagnosis and management.

The decision to focus on university hospital employees as the study population was based on several considerations. First, this group represents a diverse demographic, comprising individuals from various professional, educational, and socioeconomic backgrounds, thereby enhancing the generalizability of the findings within the local context. Second, their accessibility within a single institution allows efficient data collection using advanced diagnostic tools, such as corneal tomography and biomechanical analysis, ensuring high-quality and consistent measurements. Finally, targeting a population without prior KC diagnoses enabled exploration of subclinical cases and identification of undetected disease patterns, thereby contributing to a broader understanding of KC prevalence and associated risk factors. This approach aligns with the study's aim to inform screening and early detection strategies, which can be adapted for use in similar health care and institutional settings.

Data analysis highlighted key findings, particularly the role of corneal indices, such as the CBI, PRFI, and TBIv2, in detecting corneal abnormalities, especially in subclinical cases.^27^^,^^28^ Including indices like the TBIv2,^16^ and PRFI,^29^^,^^30^ have shown potential to enhance the detection of subclinical KC, which often remains undiagnosed by traditional methods. The TBIv2 has demonstrated improved sensitivity and specificity compared with other indices.^16^ Nevertheless, its performance may vary across populations and clinical settings, underscoring the need for further external validation. Its ability to combine both structural and functional properties makes it a valuable tool within a multimodal diagnostic approach. Together, these indices may enhance traditional methods by providing objective metrics for the early detection of corneal changes that are not readily apparent on routine examination.

The sociodemographic characterization revealed a balanced distribution between men and women, along with a homogeneous age range, thereby minimizing age-related biases in the prevalence of KC. This pattern aligns with previous studies,^31^ which emphasized the importance of homogeneous samples in diagnostic and behavioral studies. Additionally, KC was more prevalent in women, accounting for 63.1% of cases. Although some studies have reported higher rates of KC in males,^6^^,^^32^^,^^33^ many have found the opposite (or no significant difference),^34^^,^^35^ indicating that KC may affect men and women similarly. In our study, several factors may account for this imbalance. Evidence indicates that women are more likely to report illness and to seek health services.^36^ They more frequently access primary care for somatic symptoms.^37^ Women also constitute a majority among medical students in Brazil, 56.3% in a cross-sectional study at a private medical school in São Paulo^38^ and more broadly, 61.8% of all medical school enrollments nationwide.^39^ Furthermore, the availability of specialized women's health outpatient clinics in the university hospital may contribute to the overrepresentation of females observed in this sample. The analysis of behavioral variables revealed a significant association between eye rubbing and KC, with an OR of 20.34 and an RR of 9.79. These findings support the literature, which identifies repetitive mechanical trauma, such as eye rubbing, as a key risk factor for the onset and progression of KC.^29^^,^^40^ We emphasized the robust association between eye rubbing and KC, with clear interpretation of both OR and RR metrics. To support a diverse readership, we briefly explain that while the OR reflects the odds of self-reported eye rubbing among individuals with ectasia versus those with normal controls, the relative risk provides a more intuitive measure of the likelihood of ectasia in individuals who self-report eye rubbing versus those who do not.

The increase in diagnosed cases highlights the importance of multimodal diagnostic approaches. Although this study relied on a single experienced evaluator, incorporating standardized and objective diagnostic tools may reduce subjectivity in interpretation. Indices that integrate tomographic and biomechanical data have been proposed as supportive tools to improve the consistency of KC detection across different clinical settings, particularly in subclinical cases. By leveraging these advanced tools, clinicians can enhance the identification of subclinical ectatic cases and promote the adoption of unified, evidence-based diagnostic protocols across clinical settings, ultimately contributing to improved patient outcomes.^2^

Another relevant finding was the relationship between a family history of KC and diagnosis. Although this factor was less significant compared to eye-rubbing habits, it was still identified as an essential risk element in a previous study.^6^

To improve KC diagnostics, future protocols may benefit from multimodal approaches that combine corneal tomography and biomechanical assessments, with the possible support of artificial intelligence–derived indices such as TBIv2 and PRFI. Clinician training should continue to emphasize the subjective interpretation of color-coded maps while also incorporating the use and limitations of advanced metrics as supportive screening tools. Our findings illustrate how prevalence estimates vary depending on the diagnostic criteria applied. Standardizing cutoff values and promoting broader data sharing may improve consistency across studies and clinical settings, leading to more reliable prevalence data and earlier identification of KC.

Although 32.7% of eyes had TBIv2 values ≥0.43, indicating increased biomechanical susceptibility,^16^ these results should not be interpreted as reflecting the prevalence of clinical or subclinical KC. Rather, they demonstrate the potential utility of multimodal indices in screening and risk assessment. These observations reinforce the importance of early detection strategies and public awareness, particularly in populations with behavioral risk factors such as eye rubbing.

The findings have possible clinical implications. Advanced metrics, such as PRFI and TBIv2, may support more precise identification of subclinical cases that are often overlooked in conventional clinical evaluations.^2^^,^^40^

This study has limitations. First, it was conducted within a single-center population of university-affiliated individuals from a single metropolitan area, which introduces potential selection bias and limits the generalizability of the findings to broader community or population-based settings. The age of the working population at the institution did not include schoolchildren <20 years old, though it is recognized that KC often develops earlier in life. Although the inclusion of diverse professional backgrounds added some heterogeneity, the results may not fully represent the general population. Second, the cross-sectional design precludes conclusions about the temporality or progression of KC. Eye rubbing was self-reported, which may introduce information bias, and the variable was not categorized by frequency or intensity. Additionally, the risk-factor analysis was not adjusted for key confounders. Moreover, because the study population was derived from a screening program, spectrum bias may also influence the observed associations. Third, while a single experienced expert conducted all examinations to ensure consistency, the subjective component of topometric classification may still introduce some degree of interpretation bias. Fourth, although advanced diagnostic tools such as TBIv2 and PRFI were employed, longitudinal studies are needed to validate their predictive value in identifying early ectatic changes and to monitor progression over time.

Finally, a promising avenue for improving diagnostic accuracy lies in the integration of tomographic and biomechanical variables using machine learning algorithms. These analyses may offer superior classification performance by capturing nonlinear relationships and interactions among variables, thereby improving the detection of KC and related ectatic disorders.

## Conclusion

This study provides new insights into the prevalence of KC in a university hospital population in Brazil. The prevalence varied according to the diagnostic parameter applied: 13.6% of eyes (18.2% of individuals) when using the TBIv2 ≥0.8 (sensitivity 98.7%, specificity 99.2%) and 10.9% of eyes (13.8% of individuals) when using the BAD-Dv3 ≥1.9 (sensitivity 96.8%, specificity 99.3%). These findings highlight how methodological differences, particularly the use of multimodal approaches, can influence prevalence estimates. To our knowledge, this is the first study to report KC prevalence in a South American population using advanced diagnostic tools, thereby contributing to filling an essential gap in the global epidemiological understanding of the disease. Overall, our data reinforce the importance of standardized screening strategies in improving the early identification of KC. Beyond prevalence estimates, our findings also underscore the importance of public awareness strategies, particularly in populations with behavioral risk factors, such as eye rubbing.

## References

[1] Rabinowitz Y.S. (1998). Keratoconus Surv Ophthalmol.

[2] Gomes J.A., Tan D., Rapuano C.J. (2015). Global consensus on keratoconus and ectatic diseases. Cornea.

[3] Karamichos D., Escandon P., Vasini B. (2022). Anterior pituitary, sex hormones, and keratoconus: beyond traditional targets. Prog Retin Eye Res.

[4] Bui A.D., Truong A., Pasricha N.D., Indaram M. (2023). Keratoconus diagnosis and treatment: recent advances and future directions. Clin Ophthalmol.

[5] Amaral D.C., Menezes A.H.G., Vilaça Lima L.C. (2024). Corneal collagen crosslinking for ectasia after refractive surgery: a systematic review and meta-analysis. Clin Ophthalmol.

[6] Hashemi H., Heydarian S., Hooshmand E. (2020). The prevalence and risk factors for keratoconus: a systematic review and meta-analysis. Cornea.

[7] Kennedy R.H., Bourne W.M., Dyer J.A. (1986). A 48-year clinical and epidemiologic study of keratoconus. Am J Ophthalmol.

[8] Torres Netto E.A., Al-Otaibi W.M., Hafezi N.L. (2018). Prevalence of keratoconus in paediatric patients in Riyadh, Saudi Arabia. Br J Ophthalmol.

[9] de Azevedo Magalhães O., Pagano B.N., Grellmann L.V. (2024). Prevalence of keratoconus among high school students in Southern Brazil: a community-based study. Eye Contact Lens.

[10] Sriranganathan A., Chan C.C., Dhillon J., Felfeli T. (2025). Global incidence and prevalence of keratoconus: A systematic review and meta-analysis. Cornea.

[11] Martínez-Abad A., Piñero D.P. (2017). New perspectives on the detection and progression of keratoconus. J Cataract Refract Surg.

[12] Ambrósio R., Ramos I., Luz A. (2013). Dynamic ultra high speed Scheimpflug imaging for assessing corneal biomechanical properties. Revista Brasileira de Oftalmologia.

[13] Ambrosio R. (2020). Violet June: the global keratoconus awareness campaign. Ophthalmol Ther.

[14] Randleman J.B., Dupps W.J., Santhiago M.R. (2015). Screening for keratoconus and related ectatic corneal disorders. Cornea.

[15] Gomes J.A., Rapuano C.J., Belin M.W., Ambrósio R., Diseases GoPftGDPoKaE (2015). Global consensus on keratoconus diagnosis. Cornea.

[16] Ambrósio R., Machado A.P., Leão E. (2023). Optimized artificial intelligence for enhanced ectasia detection using scheimpflug-based corneal tomography and biomechanical data. Am J Ophthalmol.

[17] Correia F.F., Ramos I., Lopes B. (2022). Topometric and tomographic indices for the diagnosis of keratoconus F. https://www.ijkecd.com/doi/IJKECD/pdf/10.5005/jp-journals-10025-1018.

[18] Klyce S.D., Karon M.D., Smolek M.K. (2005). Screening patients with the corneal navigator. J Refract Surg.

[19] Ambrosio R., Salomao M.Q., Barros L. (2023). Multimodal diagnostics for keratoconus and ectatic corneal diseases: a paradigm shift. Eye Vis (Lond).

[20] Jiménez-García M., Issarti I., Kreps E.O. (2021). Forecasting progressive trends in keratoconus by means of a time delay neural network. J Clin Med.

[21] Zhang X., Munir S.Z., Sami Karim S.A., Munir W.M. (2021). A review of imaging modalities for detecting early keratoconus. Eye (Lond).

[22] Nicula C.A., Bulboacă A.E., Nicula D. (2022). Performances of corneal topography and tomography in the diagnosis of subclinical and clinical keratoconus. Front Med (Lausanne).

[23] Sedaghat M.R., Momeni-Moghaddam H., Ambrósio R. (2018). Diagnostic ability of corneal shape and biomechanical parameters for detecting frank keratoconus. Cornea.

[24] Kuo A.N., Cortina M.S., Greiner M.A. (2024). Advanced corneal imaging in keratoconus: a report by the American Academy of ophthalmology. Ophthalmology.

[25] Sedaghat M.R., Momeni-Moghaddam H., Heravian J. (2023). Detection ability of corneal biomechanical parameters for early diagnosis of ectasia. Eye (Lond).

[26] Chen X., Cao H., Huo Y. (2023). Screening of sensitive. Front Bioeng Biotechnol.

[27] Esporcatte L.P.G., Salomão M.Q., Lopes B.T. (2020). Biomechanical diagnostics of the cornea. Eye Vis (Lond).

[28] Esporcatte L.P.G., Salomão M.Q., Machado A.P., Ambrósio R. (2025). The power of predictive artificial intelligence for the detection of ectasia. Expert Rev Ophthalmol.

[29] Lopes B.T., Ramos I.C., Salomão M.Q. (2018). Enhanced tomographic assessment to detect corneal ectasia based on artificial intelligence. Am J Ophthalmol.

[30] Almeida G.C., Guido R.C., Balarin Silva H.M. (2022). New artificial intelligence index based on Scheimpflug corneal tomography to distinguish subclinical keratoconus from healthy corneas. J Cataract Refract Surg.

[31] Akowuah P.K., Kobia-Acquah E., Donkor R. (2021). Keratoconus in Africa: a systematic review and meta-analysis. Ophthalmic Physiol Opt.

[32] Papali'i-Curtin A.T., Cox R., Ma T. (2019). Keratoconus prevalence among high school students in New Zealand. Cornea.

[33] Waked N., Fayad A., Fadlallah A., El Rami H. (2011). Keratoconus screening in a Lebanese students' population. J francais d'ophtalmologie.

[34] Chan E., Chong E.W., Lingham G. (2021). Prevalence of keratoconus based on Scheimpflug imaging: the Raine study. Ophthalmology.

[35] Kanclerz P., Przewłócka K., Toprak I., Alio J. (2023). The prevalence of keratoconus in northern Poland: a cross-sectional study of patients from a primary healthcare practice. Contact Lens and Anterior Eye.

[36] Rata Mohan D.S., Jawahir S., Manual A. (2025). Gender differences in health-seeking behaviour: insights from the National Health and Morbidity Survey 2019. BMC Health Serv Res.

[37] Ballering A.V., Olde Hartman T.C., Verheij R., Rosmalen J.G.M. (2023). Sex and gender differences in primary care help-seeking for common somatic symptoms: a longitudinal study. Scand J Prim Health Care.

[38] Serinolli M.I., Novaretti M.C.Z. (2017). A cross-sectional study of sociodemographic factors and their influence on quality of life in medical students at Sao Paulo, Brazil. PLoS One.

[39] Brazil. Ministry of Health (2025). Medical Demography in Brazil 2025. Brasília: Virtual Health Library. https://bvsms.saude.gov.br/bvs/publicacoes/demografia_medica_brasil_2025.pdf.

[40] Barbara A. (2019).

