# Supplementary material for: Prevalence and Risk Factors for Keratoconus in Young Adults Assessed with Tomography and Corneal Biomechanics: A Prospective Cross-Sectional Study
Source: Ophthalmol Sci. 2026 Feb 6;6(4):101109. doi: 10.1016/j.xops.2026.101109 (PMC13019100; doi:10.1016/j.xops.2026.101109)
Supplement: Supplemental File 1 [file mmc1.pdf]

Questionnaire Link:

https://forms.office.com/Pages/ResponsePage.aspx?id=3U3TZPCvlU238Qc0pchF5ZeO

O9-qbyNDjrTbk4v6SEJUM1NFOVZFWlA5VERVR1FEODIwVjE3RVU2NS4u.

Keratoconus Screening – HUGG

Research Project on the incidence of keratoconus among HUGG staff members, using a quick, non-invasive examination (5–10 minutes) that does not require eye drops. Vision is not affected after the evaluation.

When you submit this form, it will not automatically collect personal information such as your name or email unless you provide it.

Required

1. **Name:**

2. **Sex:**

   ☐ Female

   ☐ Male

   ☐ Other

3. **Date of birth:**

4. **Do you use any eye drops?**

   ☐ No

   ☐ Yes

5. **If you answered "Yes" above, which one(s)?**

6. **Do you have any family history (grandparents, parents, siblings) of eye diseases?**

   ☐ No

   ☐ Yes

7. **If you answered "Yes" above, which condition(s)?**

8. **Do you have a history of allergies (asthma, eczema, allergic rhinitis)?**

   ☐ No

   ☐ Yes

9. **If you answered "Yes" above, which one(s)?**

10. **Have you ever been diagnosed with keratoconus?**

☐ No

☐ Yes

11. **Do you have the habit of rubbing your eyes?**

☐ No

☐ Yes

12. **Have you ever undergone eye surgery?**

☐ No

☐ Yes

13. **If you answered "Yes" above, which surgery?**

14. **Email or phone number for contact:**
