# Supplementary material for: Prevalence and Risk Factors for Keratoconus in Young Adults Assessed with Tomography and Corneal Biomechanics: A Prospective Cross-Sectional Study
Source: Ophthalmol Sci. 2026 Feb 6;6(4):101109. doi: 10.1016/j.xops.2026.101109 (PMC13019100; doi:10.1016/j.xops.2026.101109)
Supplement: Table S1 [file mmc2.pdf]

Index	Comparison Group	AUC (95% CI)	Sensitivity / Specificity (%)	Cutoff (Youden Index)	Source
**TBIv2**	Normal vs. all ectatic cases (KC + VAE-E + VAE-NT)	0.985 (0.981–0.989)	92.8 / 97.4	0.65	Ambrósio Jr. *et al.*, *AJO* 2023
**TBIv2**	Normal vs. clinical ectasia (KC + VAE-E)	0.999 (0.997–1.000)	98.7 / 99.2	0.80	Ambrósio Jr. *et al.*, *AJO* 2023
**TBIv2**	Normal vs. very asymmetric ectasia with normal topography (VAE-NT)	0.945 (0.935–0.954)	84.4 / 90.1	0.43	Ambrósio Jr. *et al.*, *AJO* 2023
**PRFI**	Normal vs. all ectatic cases	0.972 (0.967–0.977)	—	—	Ambrósio Jr. *et al.*, *AJO* 2023

Supplemental Table 1. Diagnostic Accuracy Metrics and Optimal Cut-off Values for Tomographic and Biomechanical Indices

Legend: TBIv2 (cutoff ≥ 0.8): Tomographic and Biomechanical Index version 2; KC: keratoconus; VAE-E: very asymmetric ectasia in the ectatic eye; VAE-NT: very asymmetric ectasia with normal topography; PRFI (cutoff ≥ 0.38): Pentacam Random Forest Index; AUC: area under the curve; CI: confidence interval; and ROC: receiver operating characteristic.
